# The Measurement of Strength in Children: Is the Peak Value Truly Maximal?

**DOI:** 10.3390/children8010009

**Published:** 2020-12-26

**Authors:** Hayao Ozaki, Takashi Abe, Scott J. Dankel, Jeremy P. Loenneke, Toshiharu Natsume, Pengyu Deng, Hisashi Naito

**Affiliations:** 1 Department of Sport and Health Science, Tokai Gakuen University, Miyoshi, Aichi 470-0207, Japan; ozaki-h@tokaigakuen-u.ac.jp; 2 Kevser Ermin Applied Physiology Laboratory, Department of Health, Exercise Science & Recreation Management, The University of Mississippi, Oxford, MS 38677, USA; jploenne@olemiss.edu; 3 Department of Health and Exercise Science, Rowan University, Glassboro, NJ 08028, USA; dankel47@rowan.edu; 4 Graduate School of Health and Sports Science, Juntendo University, Inzai, Chiba 270-1695, Japan; natsumetoshiharu@gmail.com (T.N.); houu.tou@gmail.com (P.D.); hnaitou@juntendo.ac.jp (H.N.)

**Keywords:** strength development, forearm muscle thickness, grip strength, ultrasonography

## Abstract

It is unclear whether the measurement of maximum muscle strength in younger children can be performed accurately due to factors such as motivation and maturity (i.e., the ability to receive instruction). If there is a large change in a ratio between muscular strength and size from the youngest to the oldest, then this might provide some indication that the youngest may not have been able to voluntarily activate their muscles for reasons mentioned previously. The purpose of this study was to observe the ratio between handgrip strength (HGS) and forearm muscle thickness (MT) across differing ages in younger children. A total of 1133 preschool children (559 boys and 574 girls) between the ages of 4.5 and 6.5 years had MT and HGS measurements and calculated the ratio of HGS/MT (kg/cm). Linear regression was used to assess the impact of age and sex on the dependent variables of MT, HGS, and the HGS/MT ratio. The HGS/MT ratio increases moderately from age 4.5 to 6.5 in both boys and girls. However, the difference in this ratio was small between the age ranges in this sample. Our results indicate children as young as 4.5 may be accurately measured with the handgrip strength test.

## 1. Introduction

Muscular strength is important for activities of daily living across the life span. During the growth process, muscle strength dramatically changes as muscle mass increases and the nervous system develops [1]. Given that most strength tests assess voluntary muscle activation, motivation, and maturity (i.e., ability to receive instruction) are important factors for accurately assessing strength. The interpolated twitch technique is often used as a method of determining the level of voluntary muscle activation in adults. However, this technique is not universal and is not particularly suitable for children. An alternative method may be to observe the relationship between strength and muscle size in children.

Handgrip strength (HGS) is often used to study strength development in children and adolescents, but most studies include children 6 years and older [2,3]. During the development process, annual changes in HGS development are not always constant in younger children (<6 years old) [3]. However, it is unknown whether this phenomenon is associated with the motivation and maturity described above. In young adults, HGS is strongly associated with muscle thickness of the forearm (MT), and the ratio of HGS to MT appears similar across different age groups (ages 20–29, 30–39, and 40–49) [4]. To our knowledge, this ratio has not been examined in children. The aim of this study was to measure the HGS and MT of children and to observe the ratio between the two across differing ages. Although some difference was expected due to development, if there was a large change in this ratio between age groups, then this might provide some indication that the youngest may not have been able to voluntarily activate their muscles for reasons mentioned previously. In addition, if age does influence the relationship between MT and HGS, then age should impact the relationship between MT and HGS (i.e., moderate the effect).

## 2. Methods

### 2.1. Participants

With the cooperation of the nursery schools and parents, 1133 preschool children (559 boys and 574 girls) between the ages of 4.5 and 6.5 years participated in this study. Children with their parents were fully informed about the purpose of the study and its safety, and informed consent was obtained from the parents for each young child. This study received approval from the Juntendo University Institutional Review Board (HSS #29-17) in accordance with the Declaration of Helsinki.

### 2.2. Handgrip Strength Measurements

Maximum voluntary HGS was measured with the right hand using a Smedley hand-dynamometer (TKK Grip-A, Niigata, Japan). All participants were instructed to maintain an upright standing position to keep their arms at their side, and to hold the dynamometer in the right hand with the elbow extended downward without squeezing. The grip span, the distance of the dynamometer’s grip bars, was adjusted to the hand size of the participants (the middle phalanx rested on the inner handle). Participants were allowed to perform one test trial followed by two maximum trials with a one-minute rest. Importantly, all participants appeared motivated during the strength tests. The highest value achieved was used for analysis. Test-retest reliability of HGS measurements using the intra-class correlation coefficient, standard error of measurement, and the minimal difference was determined for data from 10 children (5 boys and 5 girls) measured twice 24 h apart was 0.910, 0.62 kg, and 1.73 kg, respectively.

### 2.3. Muscle Thickness Measurements

Brightness-mode ultrasound images (Noblus, Tokyo, Japan) were obtained from the anterior forearm at 30% proximal of forearm length (between the styloid process and the head of the radius) on the right side of the body. The measurements were made while participants standing with the elbow extended and the forearm supinated. A linear probe with a 7.5-MHz scanning head was coated with water-based gel for ultrasound transmission and reduce pressure by the scanning head to achieve a clear image. The scanning head was placed transversely and perpendicular over on the measurement site using the minimum pressure required, and cross-sections of the forearm muscles were imaged. MT was defined as the distance between the subcutaneous adipose tissue-muscle interface and the muscle-bone interface of the ulna [4]. Further, a strong correlation (r = 0.91) has been observed between magnetic resonance imaging measured forearm flexor muscle cross-sectional area, which includes two major extrinsic flexor muscles of the fingers, and ultrasound MT [5]. Test-retest reliability of MT measurements using the intra-class correlation coefficient, standard error of measurement, and the minimal difference was determined for data from 10 children (5 boys and 5 girls) measured twice 24 h apart was 0.916, 0.22 mm, and 0.61 mm, respectively. The ratio of HGS and MT was calculated as HGS (kg) divided by MT (cm).

### 2.4. Height and Body Mass Measurements

Prior to the ultrasound measurements, standing height and body mass were measured to the nearest 0.1 cm and 0.1 kg, respectively, by using a height scale and an electronic weight scale. Body mass index was defined as body mass (kg) divided by height squared (m^2^).

### 2.5. Statistical Analysis

All statistical analyses were computed using jamovi (The jamovi project version 1.1.9.0). Linear regression was used to assess the impact of age (in months) and sex on the dependent variables of MT, HGS, and the HGS/MT ratio. In addition, an (age*sex) interaction term was created to examine if age (in months) moderated differences in MT, HGS, and the HGS/MT ratio between sexes. We also investigated, in a separate model, whether the relationship between MT and HGS (MT*age) was moderated by age (in months). Statistical significance was set to *p* < 0.05. If there was evidence of a moderating effect, we probed the interaction at low (one SD below the mean), moderate (sample mean), and high (one SD above the mean) values of the moderator.

## 3. Results

There were no age*sex interactions for MT (*β* = −0.005, *p* = 0.719), HGS (*β* = −0.026, *p* = 0.146), or the HGS/MT ratio (*β* = −0.009, *p* = 0.191). When examining the main effects of sex (*β* adjusted for age, 95% confidence interval), males had a 0.31 (0.12, 0.51) mm greater MT (*p* = 0.002), 0.68 (0.44, 0.92) kg greater HGS (*p* < 0.0001) and 0.24 (0.15, 0.34) unit greater HGS/MT ratio (*p* < 0.0001) compared to females (Table 1). There was also a main effect of age for each variable indicating that MT, HGS, and HGS/MT ratio all increased with age (all *p*-values < 0.0001; Table 1). A one month increase in age (adjusted for sex) was associated with a 0.14 (0.12, 0.16) kg increase in HGS, a 0.07 (0.06, 0.09) mm increase in MT and a 0.05 (0.04, 0.06) unit increase in HGS/MT ratio. Lastly, there was no age*MT interaction for HGS (*β* = 0.0016, *p* = 0.745). This indicates that the relationship between MT and HGS does not depend on age.

## 4. Discussion

In the present study, we observed how the ratio of HGS to forearm muscle size (MT) differed across ages in younger children. It is useful to observe the developmental status of muscle strength and function in children and adolescents [6]. Strength testing helps detect weakness in young children who may have or be at risk for neuromuscular disorders [7]. In addition, strength testing in children is also useful for identifying athletes who may have excellent abilities in sports. However, measurements for young children present some methodological difficulties. One of these methodological difficulties is the fact that it is difficult to determine whether children understand the verbal instructions given to them. In this regard, it has been pointed out that the cognitive development of 3-year-old children may not be able to fully understand the instructions regarding the generation of strength [8]. Another difficulty is to determine the level of voluntary activation (motivation) during strength tests which is an important factor in measuring maximum muscle strength. The percent voluntary activation varies by individuals, even in young adults, and has been previously reported to be approximately 95% during isometric elbow flexion [9]. In young children (<7 years old), it is very difficult to know the level of voluntary activation during maximal strength tests.

We observed the difference in HGS/MT ratio across young children and thought that, if there is a large difference in the ratio between muscular strength and size from the youngest to the oldest, then this might provide some indication that the youngest may not have been able to voluntarily activate their muscles for reasons mentioned previously. Some studies have reported HGS and muscle size development, but they were children aged 6–7 and older [10,11]. In the present study, we found that the HGS/MT ratio increases moderately from age 4.5 to 6.5 in both boys and girls. However, the difference in this ratio was small across the age range in this sample (Table 1). We also found no moderating effect of age on the relationship between MT and HGS. This supports the idea that the strength of this relationship does not depend on the age of the child. The gradual difference in the HGS/MT ratio may associate with the development of the nervous system, which supports the results of the previous studies [10,11]. A study reported that children aged 5–6 could precisely adjust the different levels of HGS production as directed by an investigator, but children aged 3 could not do so at all [8]. The results from the present and previous studies together suggested that the levels of voluntary muscle activation, i.e., motivation and maturity (i.e., ability to receive instruction) during maximal HGS measurement are similar among 4.5 to 6.5 years of age.

Our study should be interpreted with the following considerations in mind. First, we used the ratio of handgrip strength to muscle thickness to inform us about changes in voluntary activation (e.g., motivation factors) across age groups. However, it is important to consider that other measures of testing voluntary activation may not be appropriate for children. Second, our findings may be influenced to some degree by selection bias. It is possible that the children who were measured in this study might be more motivated than children who chose not to participate in this study. Finally, we measured muscle size using ultrasound estimated muscle thickness. Although muscle thickness is closely related to the cross-sectional area of the forearm flexor muscles in adults, we are not sure if the same relationship exists in children.

In conclusion, our results showed that the HGS/MT ratio increases moderately from age 4.5 to 6.5 in both sexes. However, the difference in this ratio was small across the age range in this sample. Our results indicate children as young as 4.5 may be accurately measured with the HGS test. However, additional research is needed to further explore the utility of this ratio before implementing its use broadly. For example, the use of creative but ethical approaches to minimize the possibility of selection bias will be important to either confirm or refute the use of this variable in future investigations.

## Figures and Tables

**Table 1:** The participants were divided into sub-groups according to chronological age at an interval of 3 months (0.25 years). The analysis was completed treating age as a continuous variable and this table is meant to illustrate the averages within each month for both boys and girls. The values of body mass index (BMI), handgrip strength (HGS), forearm muscle thickness (MT), the ratio of handgrip strength to forearm muscle thickness (HGS/MT), and other variables are noted as means and standard deviations (SD).

Group (Month)	*n*	Age (year)	Height (cm)	Weight (kg)	BMI (kg/m^2^)	HGS (kg)	MT (mm)	HGS/MT (kg/cm)
Boys								
54–56	52	4.6 ± 0.1	103 ± 4	16.2 ± 2.3	15.1 (1.2)	6.6 ± 2.0	23.2 ± 1.5	2.8 ± 0.8
57–59	75	4.8 ± 0.1	105 ± 4	16.5 ± 1.6	15.0 (0.8)	6.5 ± 1.6	23.2 ± 1.3	2.8 ± 0.7
60–62	68	5.1 ± 0.1	106 ± 5	17.2 ± 2.2	15.1 (1.3)	7.3 ± 2.0	23.1 ± 1.7	3.2 ± 0.8
63–65	72	5.3 ± 0.1	109 ± 5	18.0 ± 3.0	15.1 (1.5)	7.6 ± 1.9	23.5 ± 1.6	3.2 ± 0.7
66–68	59	5.6 ± 0.1	110 ± 5	18.6 ± 2.7	15.4 (1.5)	8.5 ± 2.0	24.1 ± 1.7	3.5 ± 0.8
69–71	78	5.8 ± 0.1	111 ± 4	18.7 ± 2.1	15.0 (1.0)	8.6 ± 2.1	24.3 ± 1.5	3.6 ± 0.8
72–74	67	6.1 ± 0.1	113 ± 5	19.9 ± 3.7	15.3 (2.1)	9.3 ± 2.0	24.4 ± 1.9	3.8 ± 0.8
75–77	66	6.3 ± 0.1	115 ± 5	20.6 ± 3.9	15.4 (1.9)	9.6 ± 2.2	24.5 ± 1.7	3.9 ± 0.8
78–80	22	6.5 ± 0.1	116 ± 4	20.5 ± 2.2	15.3 (1.3)	9.7 ± 2.0	24.7 ± 1.8	3.9 ± 0.7
Girls								
54–56	30	4.6 ± 0.1	103 ± 4	16.1 ± 1.9	15.1 (1.1)	6.1 ± 1.8	22.6 ± 1.4	2.7 ± 0.7
57–59	65	4.8 ± 0.1	104 ± 4	16.8 ± 2.2	15.4 (1.3)	6.4 ± 1.9	23.1 ± 1.5	2.8 ± 0.8
60–62	76	5.1 ± 0.1	106 ± 4	17.0 ± 1.9	15.0 (1.0)	6.3 ± 1.8	22.9 ± 1.5	2.7 ± 0.7
63–65	80	5.3 ± 0.1	107 ± 4	17.6 ± 2.1	15.3 (1.1)	7.1 ± 2.2	23.1 ± 1.7	3.1 ± 0.8
66–68	62	5.6 ± 0.1	110 ± 4	18.5 ± 2.7	15.1 (1.5)	8.2 ± 2.1	23.7 ± 1.9	3.4 ± 0.8
69–71	78	5.8 ± 0.1	111 ± 4	18.8 ± 2.1	15.1 (1.2)	8.1 ± 2.2	24.0 ± 1.4	3.4 ± 0.8
72–74	87	6.1 ± 0.1	112 ± 4	19.0 ± 2.4	15.1 (1.3)	8.1 ± 2.2	24.1 ± 1.7	3.4 ± 0.9
75–77	77	6.3 ± 0.1	114 ± 5	19.9 ± 2.9	15.3 (1.3)	8.5 ± 2.4	24.1 ± 1.7	3.5 ± 0.9
78–80	18	6.5 ± 0.1	117 ± 5	22.1 ± 5.3	15.9 (2.8)	9.3 ± 2.6	24.2 ± 3.3	3.8 ± 0.9

## Data Availability

The data presented in this study are available on request from the corresponding author. The data are not publicly available due to privacy restrictions.
